# Healthcare Workers’ Experiences and Views of Using Surgical Masks and Respirators, and Their Attitudes on the Sustainability: A Semi-Structured Survey Study during COVID-19

**DOI:** 10.3390/nursrep11030059

**Published:** 2021-08-07

**Authors:** Anu Venesoja, Kaisa Grönman, Susanna Tella, Salla Hiltunen, Krista Koljonen, Svetlana Butylina, Laura Rotinen, Paulus Torkki, Katri Laatikainen

**Affiliations:** 1 Department of Emergency Medicine and Services, Helsinki University Hospital, Helsinki University, 00014 Helsinki, Finland; 2 Faculty of Social Services and Health Care, LAB University of Applied Sciences, 53850 Lappeenranta, Finland; laura.rotinen@gmail.com (L.R.); Katri.Laatikainen@lut.fi (K.L.); 3 South Karelia Social and Healthcare District, 53130 Lappeenranta, Finland; 4 Department of Sustainability Science, Lappeenranta-Lahti University of Technology LUT, 53850 Lappeenranta, Finland; kaisa.gronman@lut.fi; 5 Laboratory of Separation Science, Lappeenranta-Lahti University of Technology LUT, 53850 Lappeenranta, Finland; salla.hiltunen1@gmail.com (S.H.); krista.koljonen@lut.fi (K.K.); svetlana.butylina@lut.fi (S.B.); 6 Department of Public Health, Faculty of Medicine, University of Helsinki, 00014 Helsinki, Finland; paulus.torkki@helsinki.fi

**Keywords:** COVID-19, FFP2, FFP3, healthcare (HC) workers, personal protective equipment, respirators, surgical masks, sustainability, occupational safety

## Abstract

A universal mask use was instituted in healthcare during COVID-19 pandemic in 2020. The extensive growth in the consumption of surgical masks and respirators brought new challenges. Healthcare workers had to get accustomed to wearing the facemasks continuously, raising concerns on the patient, occupational, and environmental safety. The aim of this study is to describe frontline healthcare workers and other authorities’ views and experiences on continuous use of surgical masks and respirators (facemasks) and their attitudes towards environmental and sustainability issues. A cross-sectional web-based survey was conducted in Finland during the COVID-19 pandemic in autumn 2020. The respondents(N = 120) were recruited via social media, and the data were collected using a purpose-designed questionnaire. Descriptive statistics and inductive content analysis were used to analyze the quantitative data and qualitative data, respectively. The healthcare workers perceived their own and patient safety, and comfortability of facemasks as important, but according to their experiences, these properties were not evident with the current facemasks. They considered protection properties more important than environmental values. However, biodegradability and biobased material were seen as desired properties in facemasks. Based on the results, the current facemasks do not meet users’ expectations well enough. Especially the design, breathability, and sustainability issues should be taken more into account.

## 1. Introduction

After the World Health Organization (WHO) declared the COVID-19 a global pandemic in March 2020 [1], universal masking was instructed to protect all healthcare attendees, including workers, patients, and visitors. Since then, on a daily basis, the healthcare workers began to wear surgical masks and respirators, also known as filtering face-pieces FFP2 and FFP3 and referred to in this study as a term ‘facemask’. Therefore, the need for facemasks has increased explosively [2]. In fact, 89 million facemasks are needed every month for the COVID-19 response [2].

Despite the guidelines for using Personal Protective Equipment (PPE), one-fifth of frontline healthcare workers have been infected with COVID-19 [3]. This has raised concerns in terms of the safety of the facemask users and also addressed the question of how this kind of infection is possible even if the protective facemasks have been used by the healthcare workers. According to a previous study, sufficient availability and quality of PPE have been identified as critical factors in preventing the spread of the disease [4]. Correct use and removal of PPE have also been reported to have great importance. During the repeated use and removal of PPEs, there is a risk of self-contamination or breakdown of the materials in extended use [4]. Commonly, surgical masks have protected people from the transmission of infective agents during surgical procedures. Significantly, during the pandemic, these facemasks have also been introduced to the healthcare attendees and more fitted respirators have been applied to ensure the safety of the users [5].

The effectiveness of the facemasks is controversial depending on the facemask type and how it is used [6]. The most critical properties of surgical masks and respirators are their filtration efficiency and breathing resistance. Mechanical performance, such as tensile strength and puncture resistance, must also meet certain conditions for the product to withstand wear, tear, and use. WHO has defined (COVID-19) technical specifications for personal protective equipment (PPE) and Related Inter-Agency Pharmaceutical Coordination (IPC) supplies, with regulations based on several different standards depending on the PPE type [2]. The requirements for surgical facemasks have been defined in the standard EN 14683:2019 + AC:2019, whereas respirators (FFP1, FFP2, and FFP3) follow the standard EN 149:2001 + A1:2009. For COVID-19, only FFP2- and FFP3-level respirators have been recommended.

Although the standards of the facemask requirements and properties have been well defined, users have also reported different kinds of health problems caused by the prolonged wearing periods, such as skin problems [7,8,9], breathing difficulties [10], and headaches [11]. Nevertheless, previous studies have been shown that safety is more important to facemask users than comfort [12]. There is also evidence that masks might increase contamination risks if they are not properly applied [13]. Some of these threats to HC workers could be the result of failing to adhere to recommendations and guidelines [14]. When facemask usage does cause threats to healthcare personnel, the knock-on effect is a threat to the environment [15,16].

The current value chain of the facemasks is heavily reliant on single-use masks made of plastics. Polypropylene is the most widely used material for facemasks, though polyethylene and polyethylene terephthalate have also been applied [17]. The increase in use levels of the facemasks has also increased the amount of waste and even littering, thus, causing microplastic pollution over a sustained period [18,19]. Even before the pandemic, the global waste management systems have been struggling to manage plastic waste sufficiently [20], and the rapid increase in PPE demand has made the situation even worse [21]. The amount of medical waste needing to be treated as hazardous waste due to residual pathogens [22] has been increasing manifold. For example, in a single day during the spring of 2020, 200 tons of clinical waste was produced at the epicenter of the pandemic in Wuhan, China. This is four times the capacity that the incineration facility of the city can handle [23]. Similar developments have also been reported all over the world, particularly in Europe [24] and the USA [25]. Medical waste is often disposed of by incineration in developed countries. Incineration ensures the required sterilization but leads to release of toxic gases, such as dioxin, furan, and mercury emissions in the air leading to adverse health and environmental impacts [22]. In addition, the sudden increase in medical waste has challenged the disposal capacity, and stockpiling the waste can cause long-term effects to the terrestrial and aquatic ecosystem [15].

Users’ experiences of facemasks have not been studied thoroughly, and there is an urgent need to understand these issues. The aims of this survey are to describe Finnish frontline healthcare workers’ views and experiences on continuous use of surgical masks and respirators and their attitudes towards the environmental and sustainability issues to be reflected on the existing facemask standards and requirements. To address both the user needs and environmental issues, the following questions were raised: 1. What are the healthcare workers’ views on using facemasks? 2. What kind of experiences do they have on universal use of facemasks? and, 3. How do they view facemasks in terms of environmental sustainability?

## 2. Materials and Methods

At the end of summer 2020, a multidisciplinary research team was created that included professionals and researchers from nursing, healthcare, material science, business and sustainability disciplines. The team’s objectives were to untangle the phenomenon of facemask usage and to evaluate the need for developing more sustainable facemasks in terms of environmental impact without compromising mask functionality, safety, and comfort. As a starting point, a survey of facemask users was carried out.

The target group of this survey was healthcare workers, including frontline nurses, other healthcare workers, and authorities, in particular the emergency medical services and emergency personnel, but also rescue workers, military personnel, and teachers. A web-based survey design and convenience sampling [26] were applied to achieve a wide number of descriptions from healthcare workers using facemasks. The survey design was selected to address the urgent need of exploring healthcare workers’ and other authorities’ experiences and values concerning facemask usage, which needed to be gathered swiftly in light of the ongoing pandemic. “Good practice in the conduct and reporting of survey research” was used to guide the study [27].

### 2.1. Designing the Questionnaire 

A purpose-designed questionnaire was developed to gain an understanding of HC workers’ views and experiences on appropriate surgical masks and respirators from those who have worked extensively using facemasks, together with their attitudes toward sustainability. The questionnaire was designed by a multidisciplinary research group, including nursing, medical, material science, business, and sustainability experts. The first version of the questionnaire was created and evaluated by the multidisciplinary research group and shared via a web link, giving all members the opportunity to comment on the content and visual appearance. Based on the comments, it was adjusted until a consensus was reached. After the amendment, the second version was pretested, and minor changes were made.

Altogether, the questionnaire contained 23 closed and open-ended questions. Two background questions were related to the working environment of the respondents and the frequency of the facemask usage. In turn, five open-ended questions gathered responses on experiences of facemasks in general, the mask material, their usability, views about mask reuse and recycling. Following 16 structured questions explored and prioritized the facemask users’ attitudes to the facemask properties. The Likert scale was utilized as an acceptable method for measuring self-efficacy [28]. Consequently, a four-point Likert scale was applied, categorizing the responses into four groups: 1 = not important at all, 2 = not important, 3 = important, and 4 = very important.

### 2.2. Data Collection and Setting 

This Webropol^®^ web-based survey was carried out in Finland. The data were collected in the period of one week between 28/9–6/10 2020 by sharing the questionnaire via Facebook society, which was established for frontline HC workers, including prehospital nurses, EMS personnel, emergency department workers and EMS stakeholders. Approval for the study was gained from the founder of the society. The Medical Research Act (488/1999) [29] does not require ethical review or approval for this kind of study setting.

A data protection statement and information about the research purposes were shared with the survey participants. In the study, participation acted as informed consent. Thus, answering the survey was considered consent to participate. Participation was voluntary, and the respondents received no incentives. The participants had the right to withdraw from the study by suspending their answering or by choosing not to send the answers. Accurate identifying information (e.g., name, date of birth, address or IP address, employer, or hospital district) was not collected.

The Facebook page where the survey was posted had 4253 followers at the time of the survey was carried out, and reached 4172 Likes. The Facebook page’s posts normally reach from 1000 to 40,000 subscribers. This post and survey link reached 7100 followers, 357 people opened the survey link, 198 started responding, and in the end 120 subscibers submitted their answers (Table 1).

### 2.3. Statistical Methods

Descriptive statistics were used to analyze the quantitative data. Counting was applied using IBM’s Statistical Package for Social Sciences (SPSS) version 27.0. Means, standard deviations, and distribution of responses were counted to describe the users’ values for the facemasks (Tables 2 and 4).

### 2.4. Qualitative Data Analysis

An inductive content analysis [30] was used to analyze the open-ended questions. At first, the responses were carefully read through in order to get an overall impression of the answers. The descriptions consisted of single words or short sentences. All team members had access to the data and the option to follow the progress of the analysis, increasing the reliability of the process.

Microsoft Excel was used to code and track the themes to analyze the qualitative data. In the first phase, similar descriptions received the same open code. These codes were further grouped, followed by the generation and categorizing of data into subcategories. In the final phase, subcategories were formulated into categories. For example, “I could consider reuse, if the purification was quick and environmentally friendly” was coded to “Minimizing the environmental impact of mask purification”. This code was linked to the subcategory “Minimizing environmental burden” which was merged together with another subtheme into the theme “Sustainability of production”. During the analysis, a recurrent movement occurred between the whole and the parts to keep the content open. Multiple discussions were held by the researchers to ensure reliability and credibility. In every phase, the analysis continued until the researchers reached a consensus. The last analysis phase concerned the conceptualization of the results which have been displayed in Tables 3 and 5.

## 3. Results

Most of the respondents (85.8%) worked on the HC frontline (emergency medical services, emergency departments) (Table 1). A minority of the respondents worked in other HC units (10.8%) or had another official role (3.3%). All of the respondents used facemasks weekly as a minimum, and most of them used facemasks in every shift.

### 3.1. Healthcare Workers’ Attitudes toward Facemask Properties

The HC workers assessed which facemask properties they considered the most important. The safety of users and patients was considered as most valuable property (Table 2). The facemask properties that related to users’ comfort were also seen important. However, material thickness or softness was shown to be less important.

### 3.2. Healthcare Workers and Other Authorities’ Experiences with Facemasks

The themes, subthemes, and open codes have been displayed in Table 3. Three main themes were identified affecting the facemask users’ experiences, such as low-quality breathable air developing physical symptoms, the ability to perform clinical work safely while feeling comfortable and organizational infection and quality control were identified affecting the facemask users.

#### 3.2.1. Low-Quality Breathable Air Developing Physical Symptoms

The *sensing of low-quality breathable air* appeared in descriptions of bad smell, odor intensity, feeling hot and sweaty, and also dampening during long usage. Some of the HC workers reported that they had not encountered any problems during facemask usage. However, other respondents related PPE usage to the *development of physical symptoms*. Prolonged use of PPE caused tiredness among respondents. In addition, descriptions of breathing problems, headache, skin irritation, fatigue, and various other symptoms related to the upper respiratory tract were the main physical symptoms that affected the respondent’s well-being during facemask usage.

“*During a long day at work, wearing a mask can cause all sorts of trouble, such as headaches, a burning sensation in the lungs, the rubber bands abrading behind the ears, and eyeglasses becoming foggy.*”

(R55)

#### 3.2.2. Performing Clinical Work Safely While Feeling Comfortable

Both usability and safety issues had an impact on the performance of the clinical work. Facemasks were seen as important to users’ safety. At the same time, the question was raised as to whether facemasks also create a false sense of safety. The filtration efficiency of surgical masks against microbes was the main concern to respondents. The respondents confirmed that their concentration on working tasks decreased due to the pain caused by unsuitable size or bad-fitting facemasks. Furthermore, pain and distress, and the effects on respiration, reduce the comfort of use, which may impact on quality and safety, as well as on how workers manage to perform their duties.

“*In some surgical masks, the rubber bands of ear loops are so thin that they abrade the skin, irritating the area behind the ears. The pain caused by the rubber bands reduces the concentration on work.*”

(R26)

According to healthcare workers, ensuring clear communication was not guaranteed. For example, the respondents described difficulties speaking when a mask got caught in the user’s mouth.

“*At the moment, surgical masks cannot be worn without regularly fixing their position. They keep moving all the time when talking to a patient or a colleague.*”

(R36)

The fogging of eyeglasses or visors also affected healthcare workers’ abilities to ensure clear communication. Some of the respondents did manage to avoid the fogging of eyeglasses/visors, but others did suffer from it.

“*Wearing a surgical mask with eyeglasses is difficult because you must breathe right and direct your exhale properly so that the exhaled air is not directed to the eyeglasses from underneath the top of the mask, causing them to get foggy, and then it takes a while before you can see anything.*”

(R26)

#### 3.2.3. Organizational Infection and Quality Control

Compliance with protocol and guidance was, in some cases, difficult for the HC workers. The respondents were instructed to use PPEs for all patient contact. However, the standardized facemask design and size were not always optimal for users. This led to the complication of users having to straighten out the facemask during use, as well as resorting to various assistive adaptations—for example, twisting the rubber bands.

Based on respondents’ descriptions, the quality *varies depending on the PPE provided.* As a matter of fact, quality was an ongoing issue for the respondents. For example, the facemask can easily be broken, especially during the dressing. Respondents also confirmed that their experiences of facemask quality varied depending on the manufacturer. However, it was impossible to always determine the country of origin or the raw materials used in facemasks.

“*Very much varying ‘guts’ about quality, some of them feel very high quality and safe in the face while some are like any kind of rag piece in the face.*”

(R98)

### 3.3. Healthcare Workers’ Attitudes toward Facemask Sustainability

Simultaneously, healthcare workers were asked to value the factors impacting the environmental sustainability of the facemasks. The results are shown in Table 4 and should be analyzed together with the results shown in Table 2. The answers of the healthcare workers showed a high deviation, which may reflect the complexity of the topics. The currently used plastics were not highly valued as materials and not seen as a necessity. When comparing these results to the facemask property values in Table 2, the environmental values have been considered less important than the protection properties.

### 3.4. User-Identified Factors to Improve the Environmental Sustainability of Facemasks

Based on the healthcare workers’ descriptions, three main themes and their subthemes could be identified as important for developing the facemasks to be more environmentally sustainable. The themes sustainability of production, sustainability in the use phase, and sustainability of end-of-life and their subthemes and corresponding codes have been presented in Table 5 and explained in the following chapters.

#### 3.4.1. Sustainability of Production

At the beginning of a facemask’s lifecycle, matters concerning the environmental impacts have been identified. First, healthcare workers would prefer facemasks produced in their homeland as an intrinsic value. The security of supply from domestic sources was seen as important, with locality considered to have environmental benefits thanks to shorter supply chains. Some healthcare workers raised material origin as an important factor: bio-based, recycled, and, more generally, ethically-produced materials were desired. Furthermore, some respondents took a wider view, hoping to minimize the environmental burden with responsible production processes and by optimizing lifetime greenhouse gas emissions. In addition, if facemasks are to be reused and purified, it was hoped that the environmental impacts of the purification process are to be minimized.

“*I am hoping the masks could be somehow recycled, and not cause excessive greenhouse gas emissions during production and disposal.*”

(R7)

Even if more environmentally responsible masks were hoped for, some concerns were also addressed on whether the presumably higher cost of sustainable masks would hinder procurement.

“*Hopefully, new production lines are developed and built soon! And I hope that the price does not prevent hospital districts from ordering masks that can be sustainably produced and disposed of.*”

(R77)

#### 3.4.2. Sustainability in the Use Phase

Quite naturally, the importance of safety, usability, and comfort was prioritized in the responses of healthcare workers. However, minimizing environmental impacts was also seen as important if achieved without jeopardizing mask properties and functionalities.

“*Functionality and protectiveness are the most important features. If, in addition, the material would be biodegradable/compostable or safely recyclable, it would be an added bonus.*”

(R101)

Some respondents voiced their concerns about reusable and purified facemasks, saying they would rather use single-use masks or directly refuse reusable facemasks.

“*One should not even consider reuse. Rather have them [masks] be compostable, for example.*”

(R85)

The healthcare workers also voiced that the use patterns of facemasks are varied based on different healthcare work tasks and, thus, facemask change intervals and expectations due to mask quality differ.

“*The durability of the mask has no relevance to me, as in surgical work, I must be constantly changing the mask. But in different kinds of working environment, such as in the intensive care unit, where masks are used for longer periods of time, the masks should be durable, well-fitting, and made from comfortable material.*”

(R6)

#### 3.4.3. Sustainability of End-of-Life

As noted previously, the respondents voiced both optimistic wishes and concerns towards reuse and recyclability of facemasks. One part of the respondents was satisfied by ensuring the safe disposal of masks through incineration, while some also pointed out the energy recovery possibilities of disposing of the facemasks in this way. Enabling recycling either through material recycling or recovering, the organic material, e.g., through composting, was seen as a valid development needed. However, the current infrastructure of the healthcare unit does not serve this purpose in terms of waste separation.

“*If the [mask] material could be separated after use from the mixed waste, solutions for functional separation are needed. For example, in the ambulance, there is only one trash bin and a limited amount of space.*”

(R9)

“*An enormous amount of PPE is used, so recycling/composting would be needed if it does not increase the risk of infection. Reuse would probably not be cost-efficient.*”

(R98)

The infrastructure for purification was also hoped for the reuse of the facemasks. The purified facemask should be guaranteed as safe to reuse, and, in addition, the risk of transferring pathogens via reuse should be eliminated.

“*[The respondent is hoping for] a personal multi-use mask with interchangeable filters.*”

(R93)

## 4. Discussion

In this survey study, neither the country of production nor raw material used in the PPEs manufacturing was specified. The aim of this study was to gather information about nurses, other healthcare workers, and authorities’ attitudes toward and experiences using surgical masks and respirators and how users’ experiences and attitudes reflected on the existing facemask standards and requirements. The results showed that properties (design) of facemasks could be further improved, regardless of the fact of them being highly engineered products for which standardized testing is implied. Improvement in fit, wider size range of facemasks, and use of more breathable materials are of interest and seemed like measures to prevent tiredness and headaches by experienced users.

This study showed that the safety of the healthcare workers and patients is the most valuable facemask property to healthcare workers. Their work is mentally and physically demanding even without wearing PPE. In this study, the healthcare workers described how using facemasks for long periods caused such symptoms as tiredness, pain, dry mouth, and a sense of lack of oxygen, and it was difficult to take care of patients. Overall, healthcare workers described problems experienced while using facemasks, such as discomfort, physical problems, and inadequate quality of the facemasks. These negative experiences were seen as a threat to safety. For example, focusing on the work tasks was disrupted by poor-fitting masks or when the user had to adjust the mask position with their hands, potentially leading to transmission of the disease. Although previous studies have shown that if mask users must choose between comfort and safety, then safety is the more preferable property [12].

Results conducted by Or et al. [31] and Padula et al. [32] indicated that proper training could reduce discomfort and problems with facemasks. Training is also mentioned as one key factor for preventing skin injuries [32]. When reflecting on these previous results [31,32] alongside our study results, it can be seen that there is a chance that the healthcare workers who participated in our study did not have proper training in facemask usage. Insufficient training, therefore, could explain some of the physical symptoms suffered by respondents.

The physical symptoms described by respondents were similar to those described in previous studies [7,8,9,10,11,33]. This indicates that the current facemask design and material choices are not considered enough for end-users’ comfort and well-being. Indeed, there is a need for better-designed facemasks, together with more available options regarding different sizes or masks that take into account workers with eyeglasses.

While there is an urgency to focus on the well-being and comfort of healthcare workers, there is also a need to foster sustainability in healthcare. When considering facemask sustainability, it is important to incorporate all three pillars of sustainability, i.e., economic, environmental, and social sustainability. The social pillar, which has been explored here by identifying the values of mask users, has often been overlooked by optimization studies and techno-economic assessments [21]. The environmental impacts of healthcare products can be significantly reduced by product design and guiding user behavior, as demonstrated by Jeswani and Azapagic [34]. It has been estimated that, in the USA, the healthcare sector was responsible for approximately 10% of national greenhouse gas emissions [35]. Indeed, environmental awareness among healthcare workers remains a fairly unfamiliar concern according to, e.g., Anåker et al. [36], Dunphy [37] and Sherman et al. [38]. However, this survey’s respondents indicate that understanding environmental issues was important, which aligns with the most recent results for nurses [39].

The healthcare workers raised some valid concerns about the sustainability of current medical facilities. The employees showed motivation to act more responsibly toward the environment, e.g., by preferring masks made of bio-based materials rather than fossil oil-based plastic, and through a willingness to separate waste more efficiently or to recycle or reuse the masks. However, the current infrastructure in hospitals, ambulances, and other medical facilities does not enable proper waste sorting and recycling. In addition, the current legislation prescribes incineration as the only method for dealing with medical waste materials [40,41]. Furthermore, reusing and purifying masks requires further development. According to current regulations, surgical masks and FFP2 and FFP3 respirators are defined as single-use products and, thus, the legislation does not allow for their reuse. However, several studies focus on decontamination methods, such as vaporous hydrogen peroxide treatment, ultraviolet C irradiation, or autoclave treatment, which could enable mask reuse [42,43,44]. Currently, efforts have focused on developing bio-based and biodegradable masks and respirators [45]. Even if recyclable and or reusable masks were to be developed, the sorting and logistics required for cleaning, disinfection, and storage also need to progress from the current situation.

Although biodegradable or bio-based raw materials were rated in this study as somewhat important, as well as we know, there are no commercial, bio-based, or biodegradable facemasks available for healthcare professionals. Therefore, it is difficult to compare the currently used solutions with the new alternatives yet to come. In addition, it is unclear how familiar the healthcare workers were with the material composition of the conventionally used facemasks or with the methods of the facemask decontamination and reuse. Currently, surgical masks and respirators have been handled as single-used products and incinerated after use. Nevertheless, it seems that healthcare workers were open to new solutions.

Another concern raised among healthcare workers was the cost of a more sustainable mask. It was questioned whether hospital districts would be willing to invest in better masks in terms of their sustainability both from a user and environmental point of view. A US study compared the costs of a single-use disposable N95 mask with a reusable mask, concluding that the reusable elastomeric respirators come at a lower purchase cost over a single day of patient care [46]. In addition, it was highlighted that more detailed research is needed on the costs of different mask types, including staff education and training time, alongside the costs for cleaning and disinfection. In addition, it would be important to further study the actual costs of medical staff wearing poor quality masks, which may result in lower performance at work or even sick leave. In addition, it would be important to study the effect of poor quality of masks or their improper usage (insufficient training) on the actual costs of operation of healthcare unit by taking into account expenses associated with lower work performance or sick leaves.

### Limitations

First, convenience sampling could have caused a self-selection bias [47]. It is possible that those healthcare workers and other authorities who responded to this survey represent those most unsatisfied with using facemasks. However, aiming at generalizability was not the purpose of this study. Second, the participants’ descriptions were relatively short, meaning that the web survey method did not allow for obtaining deeper descriptions concerning facemask usage and sustainability. However, there were similarities in the respondents’ descriptions. Thus, one could consider that the data was saturated in these respects. Third, the data collection via social media could see as a limitation. However, previous studies have shown that Facebook also has several advantages, for example, costs, time, and snowball effect when collecting data [48,49]. The aim of this study was to gain an overview of the facemask users’ experiences and attitudes rather than make a comparison between the users. Therefore, using social media for data collection can be seen as a valid method to collect data.

## 5. Conclusions

Based on the results, the current facemasks do not meet users’ expectations well enough. Especially, the design, breathability, and sustainability issues should be taken more into account. Overlooking the user’s experiences can jeopardize patient and healthcare worker safety and cause unexpected consequences to healthcare organizations. For example, wearing unfitting facemasks for long periods may lead to lower performance among healthcare workers, which can compromise patient and healthcare worker safety and well-being. This has the potential to cause patient safety incidents and increase healthcare workers’ falling ill and sick leaves. Research and development of a new generation of facemasks would benefit from considering the lifecycle and value chain, as well as the user experiences, meticulously. More research is needed, therefore, to assess and optimize the facemask value chain sustainability of different countries, of different operating environments, and for different use purposes in healthcare settings. Thus, healthcare professionals would be better prepared to face future epidemics without compromising their own safety or their patients.

## Figures and Tables

**Table 1:** The HC workers’ (N = 120) workplace and the frequency of using facemasks.

**Working Place**	**N = 120 (100%)**
ED personnel	21 (17.5%)
EMS personnel	82 (68.3%)
Other healthcare professionals	13 (10.8%)
Other authority	4 (3.3%)
**Frequency of facemask usage**	**N = 120 (100%)**
In every shift	114 (95%)
Weekly	6 (5%)

EMS = emergency medical services, ED = emergency department.

**Table 2:** Healthcare workers rating of the most important facemask properties from highest to lowest mean (frequency [percentage]).

	1	2	3	4	N/A	Mean	SD
Material protects the wearer	-	1 (0.8%)	18 (15.0%)	99 (82.5%)	2 (1.7%)	3.83	0.40
Easy to breathe through material	-	1 (0.8%)	21 (17.5%)	97 (80.8%)	1 (0.8%)	3.81	0.42
Material protects from splashes	-	1 (0.8%)	27 (22.5%)	92 (76.7%)		3.76	0.45
Material protects patient	-	8 (6.7%)	39 (32.5%)	72 (60.0%)	1 (0.8%)	3.54	0.62
Material does not break	-	6 (5.0%)	48 (40.0%)	66 (55.0%)		3.50	0.59
Material is odorless	-	16 (13.3%)	42 (35.0%)	62 (51.7%)		3.38	0.71
Material fibers do not come off	-	16 (13.3%)	45 (37.5%)	59 (49.2%)		3.36	0.71
Material does not cause sweating	1 (0.8%)	17 (14.2%)	56 (46.7%)	46 (38.3%)		3.23	0.72
Material does not get wet in use	-	17 (14.2%)	58 (48.3%)	44 (36.7%)	1 (0.8%)	3.23	0.68
Material is soft	-	41 (34.2%)	50 (41.7%)	29 (24.2%)		2.90	0.76
Material is thin	1 (0.8%)	66 (55.0%)	38 (31.7%)	10 (8.3%)		2.43	0.72

1 = Not important at all, 2 = Not important, 3 = Important, 4 = Very important, N/A = not available, SD = Standard Deviation.

**Table 3:** Healthcare workers’ views of wearing facemasks.

Code	Subcategory	Main Category
Experiencing odor intensity Experiencing bad smell Appearance of mask wetting Facemask feeling warm and sweaty	Sensing of low-quality breathable air	Low-quality breathable air developing physical symptoms
Sneezing and itching caused by fiber dust Developing facemask—associated nausea Developing facemask—associated headache Facemask-use causing reddening of the skin After-shift tiredness due to wearing a facemask Experiencing skin breakdown Feeling dryness of mouth Developing after-shift sore throat Higher daily duration of facemask exposure causing breathing difficulties Tiredness when wearing facemask for a long time	Developing physical symptoms	
Shortness of breath that complicates speaking Fogging of eyeglasses when facemask leaks Growing shortness of breath when taking physical efforts	Ensuring clear communication	Performing clinical work safely while feeling comfortable
Sensing of safety when wearing masks Mask fitting causing safety worries Easiness of mask use when wearing eyeglasses Feeling tightness after using facemask continually during shift Worry of infection transmission Distressing experiences of using respirators	Feeling safe and comfortable	
Facemask protocol, including universal surgical mask use Selecting a suitable mask Mask use becoming routine Delayed mealtimes causing mouth dryness Differences in opinions between healthcare workers and employers Repairing facemasks during use because of fitting problems	Compliance with protocol and guidance	Organizational infection and quality control
Rubber ear loops breaking easily Surgical masks with ribbon slow to wear Experiencing breathing difficulties depending on facemask type Being provided with masks of differing quality	Quality varies depending on provided facemask	

**Table 4:** Healthcare workers’ values regarding environmental sustainability in facemasks from highest to lowest mean.

	1	2	3	4	N/A	Mean	SD
Material is biodegradable	17 (14.2%)	30 (25.0%)	41 (34.2%)	31 (25.8%)	1 (0.8%)	2.72	1.01
Material is wood or plant-based	19 (15.8%)	38 (31.7%)	49 (40.8%)	14 (11.7%)		2.48	0.90
Material is reusable	31 (25.8%)	45 (37.5%)	29 (24.2%)	14 (11.7%)	1 (0.8%)	2.22	0.97
Material is plastic-based	74 (61.7%)	44 (36.7%)	1 (0.8%)	-	1 (0.8%)	1.39	0.51

1 = Not important at all, 2 = Not important, 3 = Important, 4 = Very important, N/A = not available, SD = Standard Deviation.

**Table 5:** Healthcare workers’ views of environmental sustainability regarding facemasks.

Code	Subcategory	Main Category
Security of supply Locality	Preferring domestic production	Sustainability of production
Biobased material Recycled material Ethically produced material	Considering material origin	
Lifetime GHG emissions Responsible production process Minimizing the environmental impact of mask purification	Minimizing environmental burden	
Considering environmental issues without compromising mask safety and usability Preferring single-use masks instead of reused	Securing mask functionalities	Sustainability in the use phase
Preventing the premature breakdown of the mask Preferring a high-quality mask when using for a longer period of time Sufficing with a lower quality mask if the mask needs to be changed frequently	Optimizing mask change interval	
Enabling mask reuse Interchangeable filters Personal masks Avoidance of transferring pathogens via reuse	Enabling safe reuse	Sustainability of end-of-life
Waste separation Material recycling Biodegradability/Compostability	Enabling recycling	
Disposing mask with incineration Recovering energy with incineration	Assuring safe disposal	

## Data Availability

The data presented in this study are available on request from the corresponding author.
